# Characterization of oscillation modes in levitated droplets using image and non-image based techniques

**DOI:** 10.1038/s41526-023-00254-7

**Published:** 2023-01-18

**Authors:** Nevin Brosius, Jason Livesay, Zachary Karpinski, Robert Singiser, Michael SanSoucie, Brandon Phillips, Ranga Narayanan

**Affiliations:** 1 University of Florida Department of Chemical Engineering, Gainesville, FL 32611 USA; 2 NASA Marshall Space Flight Center, Huntsville, AL 35812 USA

**Keywords:** Imaging techniques, Characterization and analytical techniques

## Abstract

The dynamics of levitated liquid droplets can be used to measure their thermophysical properties by correlating the frequencies at which normal modes of oscillation most strongly resonate when subject to an external oscillatory force. In two preliminary works, it was shown via electrostatic levitation and processing of various metals and alloys that (1) the resonance of the first principal mode of oscillation (mode *n* = 2) can be used to accurately measure surface tension and (2) that so-called “higher-order resonance” of *n* = 3 is observable at a predictable frequency. It was also shown, in the context of future space-based experimentation on the Electrostatic Levitation Furnace (ELF), a setup on the International Space Station (ISS) operated by Japan Aerospace Exploration Agency (JAXA), that while the shadow array method in which droplet behavior is visualized would be challenging to identify the *n* = 3 resonance, the normal mode *n* = 4 was predicted to be more easily identifiable. In this short communication, experimental evidence of the first three principal modes of oscillation is provided using molten samples of Tin and Indium and it is subsequently shown that, as predicted, an “image-less" approach can be used to identify both *n* = 2 and *n* = 4 resonances in levitated liquid droplets. This suggests that the shadow array method may be satisfactorily used to obtain a self-consistent benchmark of thermophysical properties by comparing results from two successive even-mode natural frequencies.

## Introduction

Levitation can be used to study physical phenomena in a contactless, relatively contamination-free environment. This is notably important for providing a benchmark measurement process for thermophysical properties, permitting the observation of meta-stable states of matter and highly chemically reactive materials, and studying fundamental material behavior^1^. As the name suggests, electrostatic levitation consists of a charged droplet held between two electrodes via an electrostatic field^2^. In typical processing, a material is loaded into the chamber, charged, levitated by means of a control system, and melted. After this, a variety of processing techniques can take place; in the measurement of surface or interfacial tension, for example, the droplet is subjected to an oscillatory electric field and its behavior is recorded, via a high-speed camera or other optical sensors. Using the formula derived by Rayleigh for the oscillations of an inviscid, spherical droplet^3^, one can predict the frequency at which a given waveform, or mode, will oscillate at its surface:

1 $${f}_{n}=\sqrt{\frac{n(n-1)(n+2)\gamma }{3\pi M}}$$

**Fig. 1:**
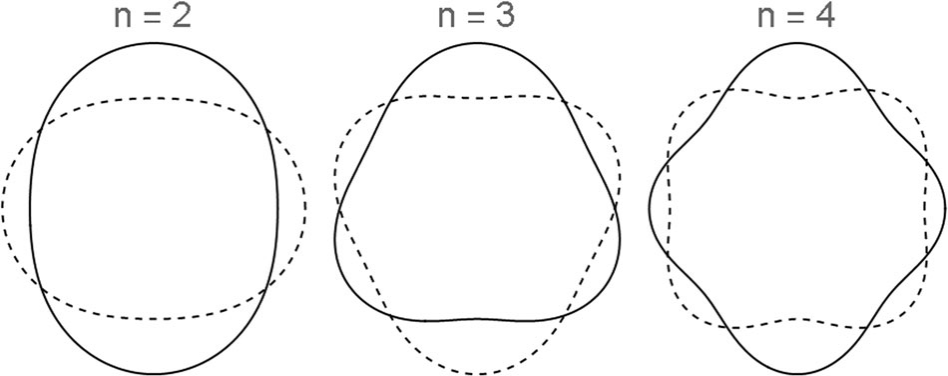
The first three modes of oscillation in an axisymmetric spherical droplet. Each mode represents the *n*th spherical harmonic and starts at *n* = 2 to satisfy the conservation of mass. The solid line represents the droplet at *t* = 0 and the dashed line represents the mode at *t* = *T*/2, where *T* is the oscillation period.

In the above equation, *γ* is the surface or interfacial tension, *M* is the mass, and *n* represents the mode of oscillation, which can be an integer from 2 to infinity. Each mode represents a unique spherical harmonic and, when the droplet is axisymmetric (independent of azimuthal angle *ϕ*), can be reduced to a Legendre polynomial with an argument of $\cos (\theta )$, where *θ* is the polar angle. Shown in Fig. 1 is a graphical representation of modes *n* = 2, *n* = 3, and *n* = 4.

**Fig. 2:**
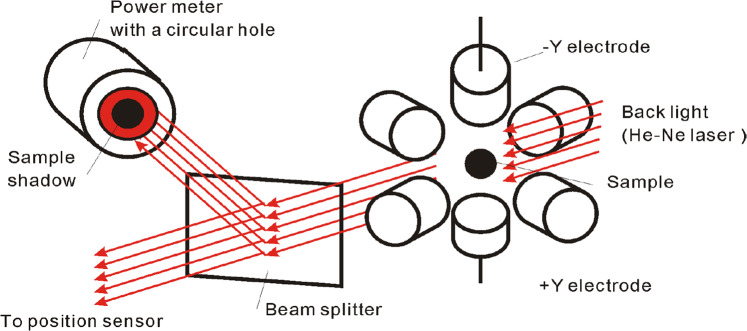
An illustration of the area array setup used to characterize the behavior of the oscillating droplet at JAXA and on ISS KIBO. The setup uses a laser to measure the amount of radiation that is blocked by the cross-sectional area of the droplet via a photosensor to infer the change in droplet shadow area from a baseline and therefore monitor droplet deformation over time (reproduced with permission from Springerⓒ (2022) from the work of ref. ^9^).

In 2016, the Japan Aerospace Exploration Agency (JAXA) launched the Electrostatic Levitation Furnace (ELF) to the KIBO module on the International Space Station for the purpose of studying materials via electrostatic levitation that are challenging to levitate on Earth. In addition to being able to test a wider variety of materials, a microgravity environment yields a more spherical droplet and thus is more theoretically tractable with analytical models that assume a spherical geometry. This setup is shown in Fig. 2 and uses a laser to cast the droplet’s shadow on a photodetector as a function of time during processing. The advantage of this detection scheme, as opposed to using a high-speed camera, for example, is that it takes less processing time to analyze the droplet’s movement when oscillated, a priority in operations on the space station.

**Fig. 3:**
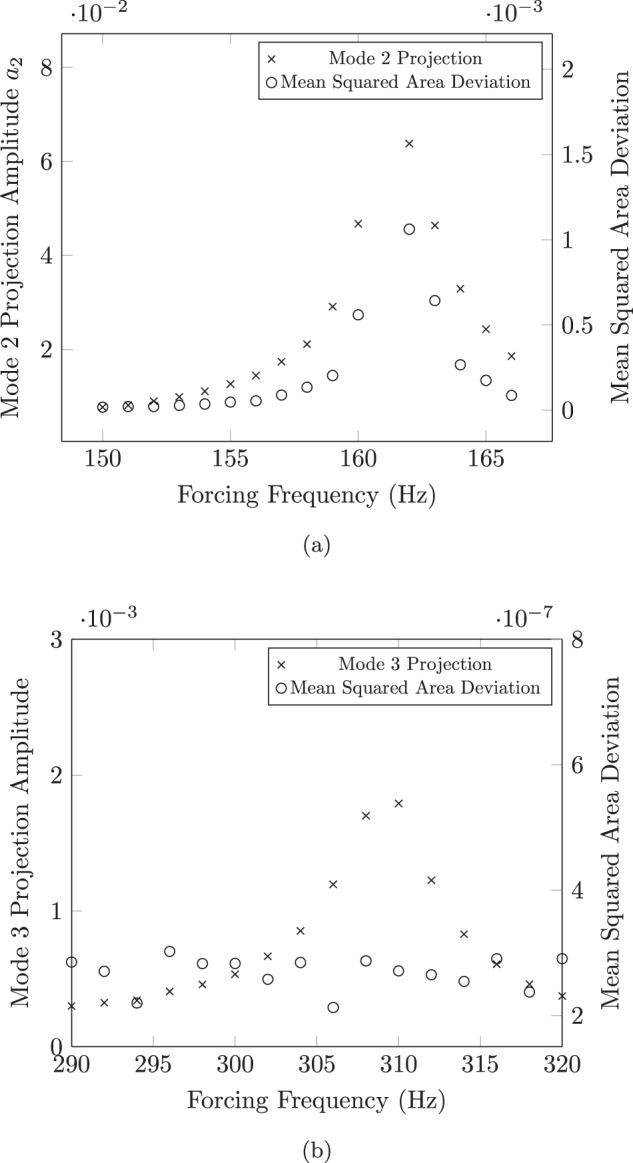
Comparison of frequency sweep analysis results obtained using an image-based method versus the simulation of a non-image-based technique for the first two oscillation modes. The resonance of **a** *n* = 2 can be easily identified via both image-based and non-image-based means, while **b** *n* = 3 resonance was only detected using the image-based technique. The sample is 57.345 mg Inconel 625 at 1350 °C. Borrowed with permission from ref. ^4^.

In a previous work by Brosius et al.^4^, it was predicted that, due to the inherent nature of this indirect method of characterizing the droplet, it would be challenging to identify odd modes of oscillation in levitated droplets on the JAXA setup—this could also be deduced from work by Egry et al.^5^. This prediction was supported by an experiment where high-speed video data was used to capture the oscillation and resonance of mode *n* = 3. Following the experiment, the magnitude of mode *n* = 3 resonance was quantified by projecting the corresponding Legendre polynomial on the droplet’s outline. Measuring the magnitude of this projection over a frequency range resulted in a clear maximum and therefore the identification of mode *n* = 3 resonance. Following this, the image data from the experiment were fed into an image processor to calculate the area of the droplet’s shadow to determine whether or not resonance could be observed using the area data alone (without the knowledge of the droplet outline itself), using a mean squared deviation of the droplet’s area for the same range of frequencies. The results of this experiment from ref. ^4^ are shown in Fig. 3 and indicate that the reduction from video data to area data removes (or at the least, reduces) the ability to identify/quantify the resonance of mode *n* = 3. For completeness, it is shown, by contrast, that the resonance of mode *n* = 2 could still be identified using non-imaging-based data.

In the following sections, a set of proof-of-concept experiments show that (1) the visualization of mode *n* = 4 is observed at expected frequencies and quantified using a spectral analysis of the droplet’s outline and (2) an “image-less" technique using only the area of the droplet can be used to identify mode *n* = 4 in addition to mode *n* = 2 and agrees with the result obtained using the outline of the droplet as the ground truth.

## Methods

### Experimental methods

All experiments in this work took place at NASA Marshall Space Flight Center (MSFC) in Huntsville, AL on the Electrostatic Levitation (ESL) Laboratory. The materials tested were Tin and Indium, chosen because of their low surface tension-to-density ratios. A detailed explanation of the process of levitation and subsequent sample processing (melting, subcooling, then imposed frequency sweeps) is given in ref. ^4^. The droplet behavior is characterized via a high-speed camera at 5000 fps at a resolution of 512 by 512 pixels for a duration sufficient to get at least 100 cycles of oscillation. Each sample was forced at 63 distinct oscillation frequencies, the number being limited by the storage capacity of the camera.

The first difference between the cited work and this work is the inclusion of a frequency sweep for finding mode *n* = 4 resonance, which followed the same regimen as modes *n* = 2 and *n* = 3. A frequency sweep is done for mode *n* = 2 at a prescribed step size, followed by mode *n* = 3 at a corresponding step size, and finally including the mode *n* = 4 frequency sweep.

**Fig. 4:**
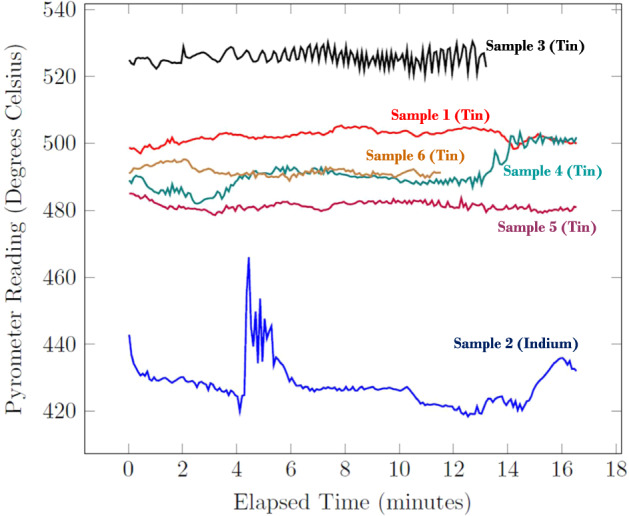
The pyrometer readings for each of the samples during experimentation. This does not include the transients involved in the melting or solidification processes at the beginning and end of processing, respectively. Recall that the pyrometer reading was used as a measure of the relative temperature difference across trials rather than an absolute measurement.

The other marked difference between the previous work and the experiments conducted for this work is the fact that the temperature measurement was not absolutely accurate for the samples due to limitations of the pyrometer that was used. Both Indium and Tin have very low melting points (156 °C and 231 °C, respectively) which were difficult to measure using the existing equipment. It was observed that the apparent melting temperature (that is, the temperature reading from the pyrometer at which the droplet was observed to melt) was much higher than realistically possible. Therefore, the sensor temperature reading was simply used to infer that the droplet maintained a constant temperature during testing—see Fig. 4. The liquid state was doubly verified by exciting the droplet in mode *n* = 2 before testing began.

The sample mass was also recorded both before and after processing. Both Tin and Indium have very low vapor pressures in the liquid state and therefore minimal mass loss (<1%) was observed.

### Analysis methods

The method in which the droplet’s shape is characterized is described in detail in ref. ^4^. In summary, it is assumed that the droplet’s deformations are axisymmetric and thus can be described by Legendre polynomials as a function of polar angle *θ*. That is, the outline of the droplet given by *r*(*θ*, *t*) is defined as:

2 $$r(\theta ,t)={R}_{0}+\mathop{\sum }\limits_{n=2}^{\infty }{A}_{n}{P}_{n}(\cos (\theta ))\cos (2\pi {f}_{n}t)$$

where *R*_0_ is the resting (non-disturbed) radius, *A*_*n*_ is the amplitude of normal mode *n*, *P*_*n*_ is the *n*th Legendre polynomial, and *f*_*n*_ are defined in Eq. (1).

In principle, the magnitude of each normal mode can be found by projecting the corresponding Legendre polynomial in polar coordinates. However, for modes of small amplitude and with slight off-axis tilt, it was more practical in this work to perform a spatial analog of a Fourier transform on the droplet outline, referred to throughout the duration of this work as the Spatial Discrete Fourier Transform, or Spatial DFT. This process allows for a slightly improved sensitivity to the identification of modes at small magnitudes.

#### Image-based processing

**Fig. 5:**
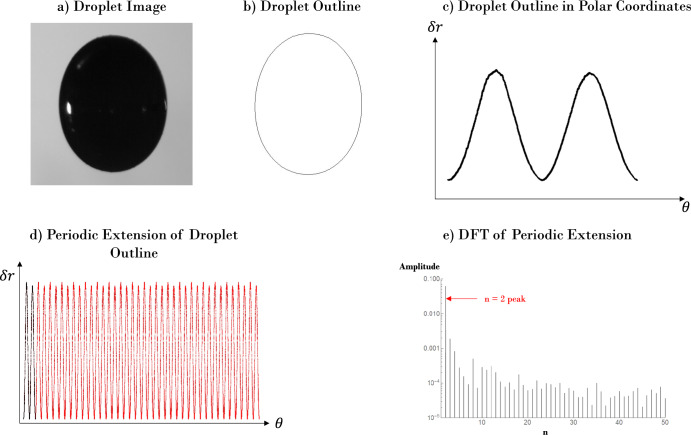
A graphic representation of the process involved in the Spatial DFT method of analyzing the outline of the droplet to identify resonance. For each image of each video, **a** the droplet image is converted into **b** an outline via image analysis software, **c** transposed from cartesian to polar coordinates about its centroid. **d** The polar form of the droplet’s outline is periodically extended 20 times and **e** a DFT is performed on the data. The amplitude of the peak corresponding to the mode of interest (in this case, mode *n* = 2) is used as the metric to quantify droplet resonance and time-averaged for each video to form the final resonance curve.

The image-based processing of the experimental data begins with the conversion of each video’s frames into a series of single-pixel-width outlines of the droplet, which are subsequently transposed into polar coordinates about the droplet’s calculated center of mass (centroid). The polar form of the droplet outline is then analyzed by taking a periodic extension (in this case 20 total cycles, or “rotations" about the center of mass, was deemed sufficient) and computing its corresponding Spatial Fourier spectrum, with each peak effectively representing discrete polar wavelengths. In other words, the DFT is used to decompose the droplet’s outline (*r*(*θ*)) into a series of sinusoidal functions of *nθ* (akin to a rectangular Fourier transform decomposing a function into sinusoidal functions of $\frac{n\pi x}{L}$, where *L* is the domain of the function of interest). Therefore, amplitude peaks resulting from the DFT correspond to each *n* and, since this process is repeated for each frame of video, are recorded as a function of time. The overall process for each frame is shown in Fig. 5.

The time-averaged amplitude of each mode is then computed for every frequency tested on the droplet to fully capture the so-called “resonance curve” for a given mode. Denoting the amplitude for each peak as *α*_*n*_(*t*), and assuming its behavior can be described by ${\alpha }_{n}(t)={a}_{n}\sin (2\pi {f}_{n}t)$ (where *f*_*n*_ is given by Eq. (1)), one can approximate the time-averaged modal amplitude *a*_*n*_ given *N* frames with the below formula:

3 $${a}_{n}=\sqrt{2}{\left(\frac{\int\nolimits_{0}^{T}{({\alpha }_{n}(t))}^{2}\mathrm{dt}}{\int\nolimits_{0}^{T}\mathrm{dt}}\right)}^{\frac{1}{2}}\approx {\left(\frac{2}{N}\mathop{\sum }\limits_{i = 1}^{N}{a}_{n,i}^{2}\right)}^{\frac{1}{2}}$$

where *T* is the total period of recording time for a given forcing frequency.

It has previously been shown in the density measurement of levitated materials^6^ that selection of the centroid as the origin can result in reduced stability of fit and (in the case of this work) variation in the absolute amplitude of the spatial coefficients *α*_*n*_. Since the relative amplitude is of concern, this will not impact the findings of this analysis (i.e., what is deemed the natural frequency for a given mode). Nevertheless, it would be a valuable investigation to understand the implications of the placement of the origin.

#### Generation and subsequent analysis procedure of simulating area array data using video data

**Fig. 6:**
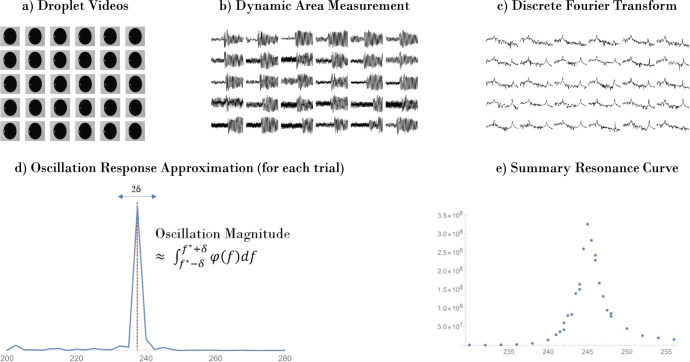
A graphic representation of the process of simulating and subsequently analyzing non-image-based data using pre-existing image-based data. In this process, **a** the videos of the droplet’s oscillation behavior at set forcing frequencies are **b** converted into area vs. time data, **c** broken down into frequency components via a DFT and **d** each dataset is quantified by integrating a frequency “band" centered around the forcing frequency. **e** The resonance curve reflects this amplitude versus the forcing frequency and shows a clear peak, in this case for mode *n* = 4.

Following the procedure of ref. ^4^, the video data is transformed to area data to simulate the observation of the drop with a shadow array rather than a camera. Image analysis software was used to convert the video to binary pixel images and calculate the area of the droplet for each frame. These data are subsequently analyzed to quantify excitation of the mode of interest. Due to the significantly reduced amplitude of oscillation upon mode *n* = 4 resonance, the area oscillations immediately about the forcing frequency were isolated by means of a DFT. This general procedure is outlined graphically in Fig. 6.

To summarize Fig. 6, each of the videos acquired during the mode *n* = 4 frequency sweep are batch processed to transform from image data to droplet area versus time. A DFT is then applied to each dataset, and the spectrum immediately surrounding the forcing frequency is integrated to find the approximate response amplitude. This approach effectively isolates the droplet’s area change as it relates to the frequency at which it was forced and therefore reduces the noise imparted by other non-resonance-based oscillations. Plotting the amplitude of response versus the frequency of forcing for a frequency sweep yields the familiar resonance curve using the area data alone.

### Reporting summary

Further information on research design is available in the Nature Research Reporting Summary linked to this article.

## Results and discussion

### Observation of *n* = 2, *n* = 3, and *n* = 4 modes using the image-based approach

**Fig. 7:**
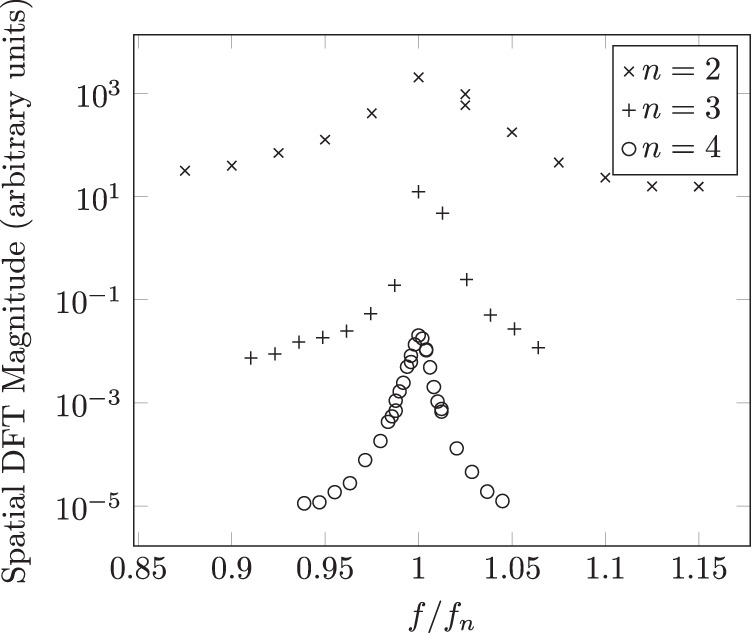
The resonance curves for each normal mode as a function of the ratio of the forcing frequency to the experimentally observed resonant frequency. Sample is 60.4 mg Tin.

A summary of the experiments is shown in Table 1. Six total samples (five Tin, one Indium) were successfully processed; that is, clear resonance was observed for each of the *n* = 2, *n* = 3, and *n* = 4 modes. Furthermore, it can be observed that the ratio of the modal frequencies are consistent with what is predicted by Eq. (1). A graphical representation of this self-consistency is shown in Fig. 7, where the amplitude of response for each mode is plotted as a function of the ratio $\frac{f}{{f}_{n}}$, where *f*_*n*_ is the experimentally observed resonant frequency of mode *n*.

**Table 1:** A summary of the experimental results, identifying the frequency at which the droplet was observed to oscillate most strongly for modes *n* = 2, *n* = 3, and *n* = 4 for purposes of comparing with their theoretical frequency ratios predicted by Eq. (1).

Sample	Material	Mass (mg)	*f*_2_ (Hz)	*f*_3_ (Hz)	*f*_4_ (Hz)	*f*_3_/*f*_2_	*f*_4_/*f*_2_	*f*_4_/*f*_3_
1	Tin	50.3	85 ± 2	172 ± 2	270 ± 2	2.02 ± 0.05	3.18 ± 0.08	1.57 ± 0.02
2	Indium	39.8	104 ± 2	195 ± 2	305 ± 1	1.88 ± 0.04	2.93 ± 0.06	1.56 ± 0.02
3	Tin	50.4	91 ± 1	174 ± 1	273 ± 1	1.91 ± 0.02	3.00 ± 0.03	1.57 ± 0.01
4	Tin	66.7	78 ± 2	149 ± 2	234 ± 1	1.91 ± 0.06	3.00 ± 0.08	1.57 ± 0.02
5	Tin	60.4	80 ± 2	156 ± 2	245 ± 0.5	1.95 ± 0.05	3.06 ± 0.08	1.57 ± 0.02
6	Tin	60.3	84 ± 2	158 ± 2	245 ± 1	1.88 ± 0.05	2.92 ± 0.07	1.55 ± 0.02
Theoretical values						1.94	3	1.55

### Proof of concept using simulated area array data

Using video data to create a simulation of the data obtained using a shadow array approach (a method to be employed in future testing on the ISS in the shadow array setup shown in Fig. 2), it was shown herein that it is still feasible to isolate and quantify the resonance of mode *n* = 4 by performing DFT analysis on the corresponding area versus time data. To this end, the so-called “benchmarking" method described in ref. ^4^ can be performed even if odd modes (*n* = 3) are difficult to observe with a shadow array setup.

**Fig. 8:**
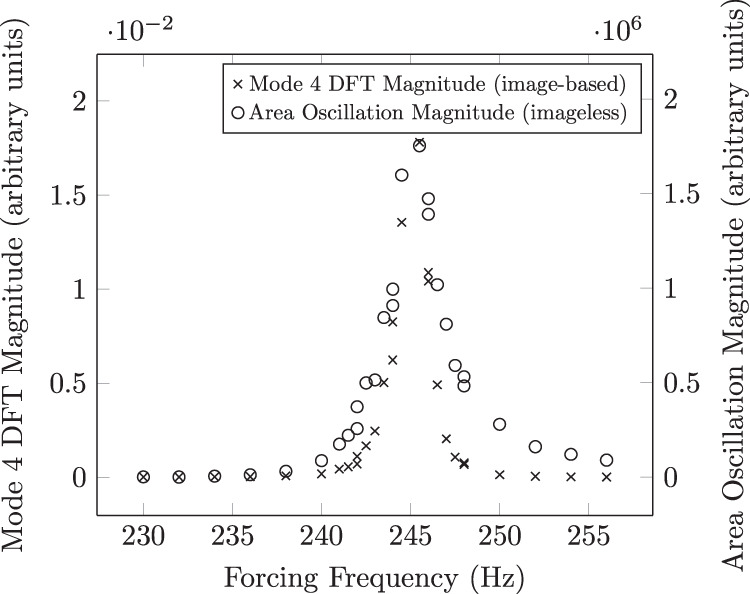
The comparison between the resonance quantification of mode *n* = 4 using an image-based method and an image-less method. Sample is 60.4 mg Tin.

The proof of concept is shown in Fig. 8. It can be observed that the frequency at which the measured area oscillation response is a maximum corresponds directly with the frequency at which the video data showed the strongest *n* = 4 oscillation amplitude. In other words, the quantification methods for both image-less and an image-based approach agree in the case of *n* = 4, as it had been shown in prior works with *n* = 2.

### Accuracy and precision of measurement

A thorough commentary on precision and accuracy as it concerns the general procedure of reporting natural frequency is documented in ref. ^4^. To summarize, the precision (that is, the reporting on the natural frequency for a given sample and mode) is primarily driven by the step size taken during the experiment. In other words, if the step size is 1 Hz and the amplitude clearly peaks at 185 Hz, the reported value is 185 ± 1 Hz. Therefore, the experiment can be carried out with theoretically arbitrary precision.

The accuracy of the measurement (which is defined as the possible shift in resonant frequency from the ‘actual’ natural frequency of the mode) can be impacted by several things that depend on experimental parameters, material properties, and operating environment. An impressively thorough analysis of imaging-related errors in the levitated droplet environment is presented in ref. ^7^ and includes uncertainty propagation due to edge detection, pixel volume, and aspect ratio. The primary drivers of experimental error associated with reporting the resonant frequencies of a forcibly oscillated droplet are (1) the electric field, (2) the gravitational field, and (3) the amplitude of deformation while the droplet has forcibly oscillated. Each of these sources of error are managed by either minimizing their effect or referring to theoretical works that quantify the related error, which has been postulated to be on the order of 1% but realistically no more than 5% of the reported natural frequency^4^.

Notably, in addition to these aforementioned primary sources of measurement error, there is also a possible impact to measurement accuracy (there is a resonant frequency shift) if the droplet is spinning while being forcibly oscillated. During experimentation, extreme care was taken to monitor the spin of the droplet by monitoring high-definition live video with which one could easily infer the rotation of the droplet during the melting process and subsequently afterward by watching the recorded high-speed video of the forced oscillations. If the droplet was rotating during the oscillation for any of the trials, that trial was discarded and another oscillation at that frequency was attempted. The error associated with droplet rotation can also be reported in the form of a frequency shift and has been thoroughly investigated by many researchers, notably in ref. ^8^, wherein the natural frequency of a rotating droplet through linear stability analysis was presented as

4 $$f\approx {f}_{0}\left(1+\frac{19}{21}\frac{{{{\Omega }}}^{2}}{{\omega }_{0}^{2}}\right)$$

where *f*_0_ is the natural frequency of a non-rotating droplet according to Eq. (1), Ω is the rotational angular frequency, and *ω*_0_ = 2*πf*_0_. The rotational frequency of the droplets tested in this work did not exceed ≈1 Hz as evidenced by high-speed video analysis and constant monitoring of the live-stream high-definition video feed of the experiment. For the most conservative (low frequency) case of mode *n* = 2 with a natural frequency of 78 Hz, a 1 Hz rotation frequency would result in an error of ≈0.01%.

### Concluding remarks

In summary, the *n* = 2 and *n* = 4 oscillation modes can be detected via an area array setup as is available on the ISS KIBO module’s ELF facility. This assertion is supported by the agreement between the actual image-based data and the simulated area array data. The Spatial DFT method in which the droplet’s shape was analyzed varies slightly from the previously used Projection Method but is robust in its ability to detect oscillating modes that are slightly off-axis and small amplitude.

The so-called “odd"-modes of oscillation remain to be elusive in an image-less approach, due to the fact that, for a nearly axisymmetrical oscillating droplet, the odd Legendre polynomials integrate to zero (or, in reality, a vanishingly small value) when compared to their even counterparts. This can potentially be resolved, as alluded to in ref. ^5^, by viewing the droplet from different viewing angles with respect to the direction of the oscillating electric field.

Ultimately, this work will serve to facilitate the benchmarking objectives outlined in ref. ^4^ where a sample can be levitated and multiple resonance points can be calibrated with theoretical predictions to add additional precision to the measurement of surface tension in microgravity environments.

## Data Availability

All relevant data are available from N.B.

## Supplementary informat

Reporting Summary

## Code availability

All relevant codes for image analysis protocols are available from N.B.
